# Clinicopathological characteristics of young never smoker females with oral cavity squamous cell carcinoma: A STROBE compliant retrospective observational study

**DOI:** 10.1097/MD.0000000000023871

**Published:** 2021-02-05

**Authors:** Minsu Kwon, Dong Kyu Lee, Seung-Ho Choi, Soon Yuhl Nam, Sang Yoon Kim, Yoon Se Lee

**Affiliations:** a Department of Otorhinolaryngology-Head and Neck Surgery, Korea University Anam Hospital, Korea University College of Medicine; b Department of Otolaryngology, Asan Medical Center, University of Ulsan College of Medicine, Seoul, Republic of Korea.

**Keywords:** female, never smoker, oral cavity squamous cell carcinoma, young adult

## Abstract

Although the incidence of oral cavity cancer (OCC) in young never smoker females is increasing worldwide, there has been little research on the etiologies and characteristics of these patients to date. In this study, we sought to evaluate the annual increase in OCC incidence in young never smoker females (YNSF) in our hospital as well as to investigate their clinicopathological characteristics and different disease courses compared with those of other OCC patients. We retrospectively reviewed the medical records of patients who were diagnosed and treated at our tertiary referral hospital from 2006 to 2016. The annual incidence of OCC and proportion of YNSF (never smoker females aged 45 years or younger at the time of diagnosis) among the enrolled OCC patients were evaluated. The characteristics and prognosis of the YNSF group were analyzed using their clinicopathological and survival data. Among the OCC patients primarily enrolled in this study, the proportion of YNSF did not show significant annual increase. There were 32 YNSF among 354 OCC patients (9%), who were ultimately included for the analyses of clinicopathological characteristics and survival. However, YNSF showed no significant differences compared with other OCC patients, even in subgroup analyses for overall survival. Our study did not demonstrate significant changes in the annual proportion of YNSF among OCC patients. In addition, differences in neither clinicopathological characteristics nor survival were noted between YNSF and other OCC patients.

## 1 Introduction

Head and neck cancer (HNC) develops with increasing age and tobacco and alcohol use.^[1,2]^ However, despite gradual decline in the smoking population over the recent decade, there has been an increase in the number and proportion of patients with oropharyngeal cancer, particularly in the non-smoking young population. Epidemiologists have identified human papillomavirus (HPV) as a new and rampant cause of and a significant prognostic marker for oropharyngeal cancer.^[3,4]^ Changing incidence trends, risk factors, and HNC-affected groups must be carefully monitored for proper diagnosis and management, including prevention.

Recently, there has been an increase in the number of young HNC patients, especially those with oral cavity cancer (OCC), in the United States, which is interesting since young age is weakly correlated with classical risk factors including smoking and drinking.^[5,6]^ OCC in young non-smokers is presumed to be mainly caused by HPV infection and genetic mutations; however, conclusions regarding the associations of these factors with OCC genesis remain inconsistent.^[7–10]^ In terms of prognosis, some studies reported that OCC in young patients showed more aggressive tumor features and led to worse survival, whereas other studies demonstrated no difference in prognosis between young and old patients.^[10–15]^

In young HNC patients, the number of females with OCC is increasing rapidly, despite a majority being non-smokers.^[16,17]^ Perhaps, there is a bias toward a lower frequency and duration of exposure to tobacco in young individuals and females. Nevertheless, the increasing number of young never smoker females (YNSF) with OCC is a worldwide phenomenon.^[18]^ HPV infection, as a known cause of oropharyngeal cancer, was initially believed to be the cause of OCC in young females; however, the etiology remains unclear as there has been no reduction in the incidence or improvement in the survival rate in young females despite the introduction of HPV vaccination.^[16]^ In an old study, OCC in YNSF showed worse prognosis, subsequently requiring aggressive treatment, but the evidence was not strong.^[19]^

Although the incidence of OCC in YNSF is increasing worldwide, there has been little research on the etiologies and characteristics of these patients so far. Consequently, in this study, we aimed to evaluate annual increase in the proportion of YNSF with OCC in our single tertiary referral hospital in Far East Asia. Moreover, we sought to investigate clinicopathological characteristics and determine any differences in disease courses between the studied population and other patient groups in order to identify specific risk factors.

## 2 Methods

### 2.1 Patients

**Figure 1:**
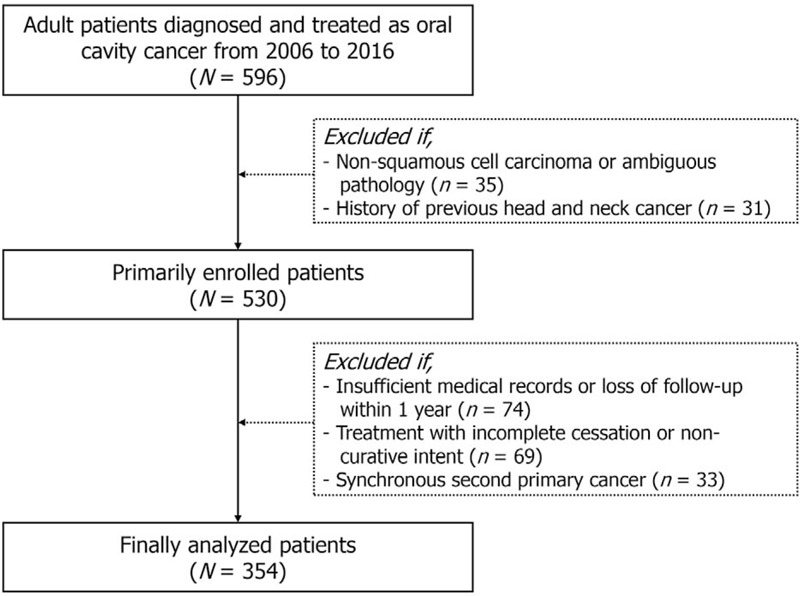
Diagrammatic representation of patient enrollment.

We retrospectively reviewed the medical records of patients who were diagnosed and treated at our tertiary referral hospital from 2006 to 2016. Only adult patients (age, ≥18 years) at the time of diagnosis were included in the analyses, and patients with recurrent cases or primary tumor with non-squamous cell carcinoma origin were excluded. Patients who did not receive definite initial treatment with curative intent or who were lost to follow-up within 1 year were also excluded. Additionally, patients with a history of HNC or synchronous second primary cancer (SPC) at the initial diagnosis were not included in the analysis. YNSF was defined as a never smoker female OCC patient aged 45 years or younger at the time of diagnosis, according to the report by Toporcov and colleagues in 2015.^[18]^ We determined the annual proportion of OCC patients enrolled at our institution during the study period and analyzed increase in this proportion. The process of inclusion for analyses is summarized in Figure 1.

### 2.2 Ethical statement

This study was conducted under following approval of the institutional review board (IRB No. S2020-0074-0001) of Asan Medical Center and conducted according to the guidelines of the Declaration of Helsinki. Patient consent was waived due to the retrospective study design.

### 2.3 Variables

Demographics of all patients, such as sex, age, smoking and drinking history, body mass index (BMI), comorbidities, and family history of cancer, were investigated. The smokeless (chewing or dipping) tobacco is seldomly used in our country, hence, the general definition of “tobacco use” is applied to the concept equivalent to “smoking.” Medications such as steroids or immunosuppressants that could affect the anticancer immunity were also considered. The oral hygiene status of the patients was evaluated, and the stages of hygiene were classified according to a dental examination routinely performed during initial staging work-up in preparation for radiotherapy (RT). We defined “poor” oral hygiene in this study as the case where the patient's dental condition would be necessary to receive active treatment assessed by a dentist. Pretreatment characteristics of the primary tumor, including subsite and tumor–node–metastasis classification according to the 8th edition of American Joint Committee of Cancer staging manual, as well as neutrophil–lymphocyte ratio (NLR) and platelet–lymphocyte ratio (PLR), which are regarded as prognostic markers for HNC, were evaluated.^[20,21]^ Finally, pathological data including depth of invasion (DOI), perineural invasion (PNI), lymphovascular invasion (LVI), and extranodal extension (ENE) were collected from surgical specimens.

### 2.4 Treatment and follow-up

All patients underwent initial surgical treatment with curative intent. Treatments were performed based on the guidelines of the National Comprehensive Cancer Network according to the patient's stage, and RT or chemoradiotherapy (CRT) was subsequently introduced as an adjuvant treatment according to the postoperative pathology.^[22]^ Patients were followed up, and recurrence or SPC development was closely monitored according to the schedule planned after the initial treatment. The final status and survival of patients at the time of the last follow-up were also investigated.

### 2.5 Statistical analyses

Clinicopathological characteristics and prognosis of the YNSF group were analyzed based on the aforementioned variables. For comparison between groups, chi-square tests (or Fisher's exact tests) and student *t* tests (or Mann–Whitney *U* tests) were used for categorical and continuous variables, respectively. Survival rates of the YNSF and other groups were compared using Kaplan–Meier survival plots and log rank tests. Statistical analyses were performed using IBM SPSS (ver. 22.0; IBM Corp, Armonk, NY), and statistical significance was defined as two-sided *P* < .05.

## 3 Results

### 3.1 Patient demographics and annual YNSF proportion

**Figure 2:**
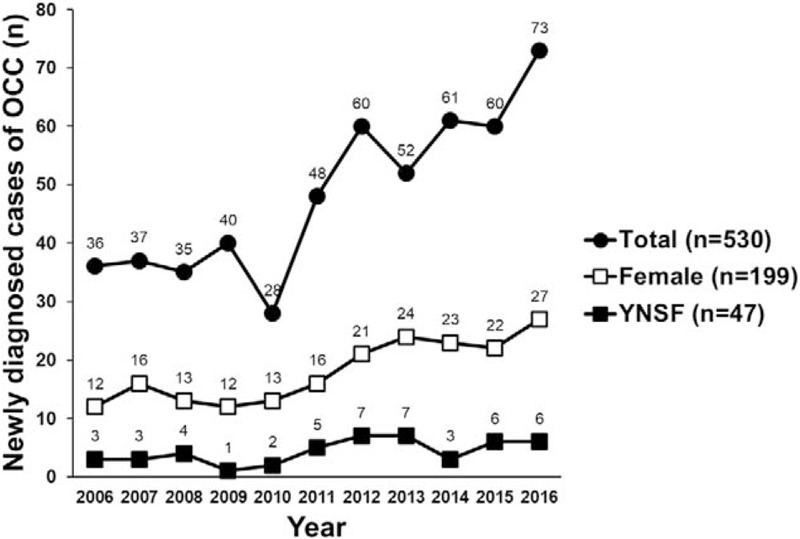
Annual incidence of newly diagnose cases of oral cavity cancer visiting our hospital from 2006 to 2016. OCC = oral cavity cancer, YNSF = young never smoker females.

The number of OCC patients initially enrolled in this study was 530. Of these patients, 199 (37.5%) were females and 47 (8.9%) were YNSF. There was an annual increase in number of newly diagnosed patients with OCC and of female patients, although the proportion of YNSF did not increase significantly. Rather, the proportion of YNSF decreased (Fig. 2). Next, the clinicopathological characteristics of the 354 patients ultimately included were analyzed. Median patient age was 54 (21–87) years, and 214 (60.5%) patients were males, 140 (39.5%) females, and 32 (9%) YNSF. There were 175 (49.4%) never smokers, and 8 (2.3%) patients were receiving steroids or immunosuppressants. Overall, 104 (29.4%) patients presented a family history of cancer, and 140 (39.5%) showed poor oral hygiene before treatment. The most common primary tumor site was the tongue (284 patients, 80.2%), and 147 (41.5%) patients were diagnosed with advanced stage III/IV cancer at initial presentation. All patients underwent radical surgery, and 150 (42.4%) patients received postoperative RT or CRT. During the median follow-up period of 50.9 (12–207.6) months, OCC recurrence was noted in 93 (26.3%) patients and 53 (14.9%) patients died. Detailed clinicopathological characteristics are summarized in Table 1.

**Table 1:** Patient characteristics (N = 354).

Characteristic	N (%) (unless otherwise indicated)
Age, years, median (range)	54 (21–87)
Sex, male/female	214 (60.5)/140 (39.5)
BMI, kg/m^2^ (mean ± SD)	23.8 ± 3.5
Smoking, never/ex-/current	175 (49.4)/55 (15.5)/124 (35)
Alcohol, never/social/heavy	22 (6.2)/245 (69.2)/87 (24.6)
Steroids or immunosuppressants	8 (2.3)
ECOG PS, 0/1/2/3	326 (92.1)/24 (6.8)/3 (0.8)/1 (0.3)
Marital status, engaged	307 (86.7)
Family history of cancer, present	104 (29.4)
Dental status, normal/poor/unknown	134 (37.9)/140 (39.5)/80 (22.6)
NLR, median (range)	1.9 (0.5–12.7)
PLR, median (range)	121.5 (12.7–501.6)
Site, tongue/FOM/buccal mucosa/other^∗^	284 (80.2)/26 (7.3)/20 (5.6)/24 (6.8)
Tumor stage, T1/T2/T3/T4^†^	138 (40)/126 (35.6)/73 (20.6)/17 (4.8)
Node stage, N0/N1/N2/N3^†^	241 (68.1)/48 (13.6)/36 (10.2)/29 (8.2)
Overall stage, I/II/III/IV^†^	120 (33.9)/87 (24.6)/75 (21.2)/72 (20.3)
Pathologic findings	
Resection margin, positive	18 (5.1)
Depth of invasion, mm, median (range)	6 (0.5–35)
Lymphovascular invasion, present	43 (12.1)
Perineural invasion, present	52 (14.7)
Extranodal extension, present	29 (8.2)
Treatment	
Surgery only	204 (57.6)
Surgery + PORT or CRT	150 (42.4)
Follow-up information	
Duration, months [median (range)]	50.9 (12–207.6)
Recurrence, present	93 (26.3)
SPC, present	16 (4.5)
Last status, NED/AWD/DOD/DOC	283 (79.9)/18 (5.1)/50 (14.1)/3 (0.8)
Cause of death, OCC/SPC/non-cancerous	50 (14.1)/2 (0.6)/1 (0.3)

### 3.2 Comparisons of characteristics between YNSF and other OCC patients

We sought to examine the presence of parameters specific to the YNSF group among the aforementioned clinicopathological characteristics of OCC patients. BMI of the YNSF group was lower than that of the rest of the patients (21.5 ± 2.2 vs 24 ± 3.5 kg/m^2^, *P* = .012); however, there were no differences in terms of family history of cancer or oral hygiene status (all *P* > .05). In addition, there were no differences in NLR (2.6 ± 1.6 vs 2.3 ± 1.4, *P* = .327) or PLR (145.9 ± 45.8 vs 131.7 ± 57.5, *P* = .292) between the two groups. In the comparative analysis of primary OCC characteristics, there were no significant differences in terms of the most frequent subsite and initial overall stage between the two groups (all *P* > .05). Mean DOI was 8.9 ± 6.1 mm in the YNSF group and 7.7 ± 6.2 mm in the rest of the patients, and this difference was not significant (*P* = .991). Cervical lymph node metastasis was identified in 11 (34.4%) of the YNSF patients and 102 (31.7%) of the remaining patients (*P* = .843). Finally, adverse events including PNI, LVI, and ENE did not differ between the two groups (all *P* > .05, Table 2).

**Table 2:** Comparison of clinicopathological characteristics between young never smoker females and other patients with oral cavity cancer.

Characteristic	YNSF (n* *= 32)	Other (n* *= 322)	*P*
BMI, kg/m^2^ (mean ± SD)	21.5 ± 2.2	24 ± 3.5	.012
Family history of cancer, present	10 (31.3)	94 (29.2)	.84
Dental status, poor	9 (28.1)	131 (40.7)	.188
NLR (mean ± SD)	2.6 ± 1.6	2.3 ± 1.4	.327
PLR (mean ± SD)	145.9 ± 45.8	131.7 ± 57.5	.292
Site, tongue	28 (87.5)	256 (79.5)	.356
Tumor stage, III/IV	11 (34.4)	79 (24.5)	.286
DOI, mm (mean ± SD)	8.9 ± 6.1	7.7 ± 6.2	.991
Perineural invasion, present	7 (21.9)	45 (14)	.29
Lymphovascular invasion, present	1 (3.1)	42 (13)	.152
Node stage, N+	11 (34.4)	102 (31.7)	.843
Extranodal extension, present	3 (9.4)	26 (8.1)	.736

### 3.3 Comparisons of survival rates between YNSF and other OCC patients

**Figure 3:**
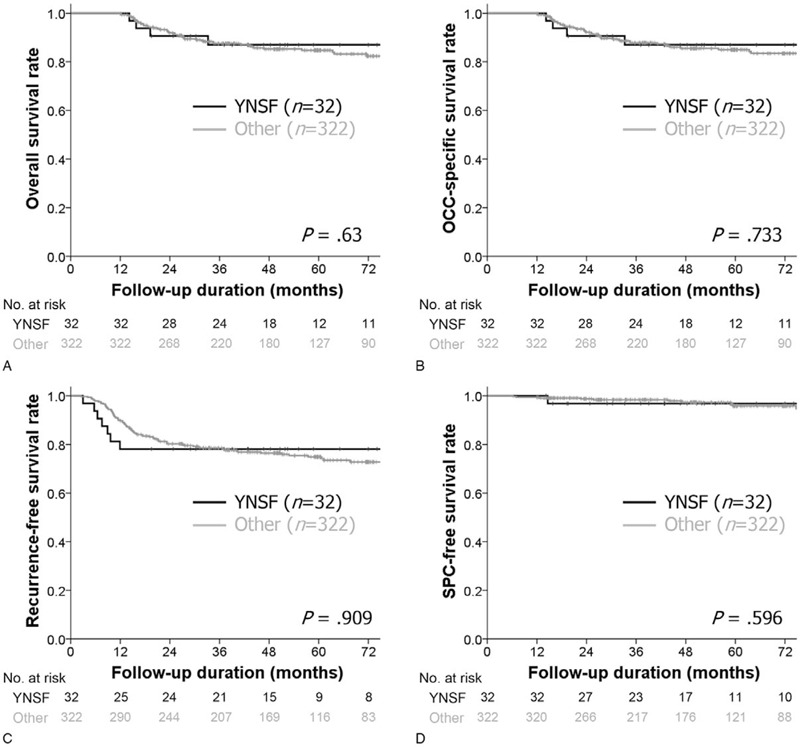
Comparisons of (A) overall, (B) oral cavity cancer (OCC)-specific, (C) recurrence-free, and (D) second primary cancer (SPC)-specific survival rates between young never smoker females (YNSF) and other OCC patients using Kaplan–Meier survival plots with log rank test.

**Figure 4:**
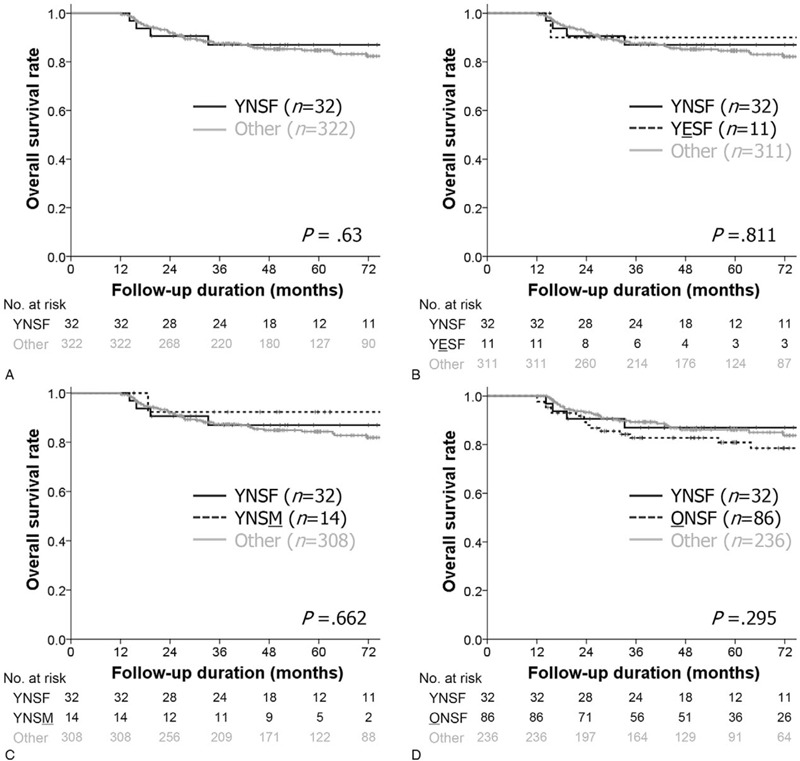
Comparisons of overall survival rates between young never smoker females (YNSF) and (A) other oral cavity cancer patients and subgroup analyses among (B) YNSF, (C) young never smoker males (YNSM), and (D) old never smoker female (ONSFs) using Kaplan–Meier survival plots with log rank test.

We next examined the prognostic characteristics of YNSF patients through cause-specific survival analyses. The 5-year overall survival (OS) rate was 90.5% in the YNSF group and 87.2% in the other group, and there was no significant difference between the two groups (*P* = .63). In addition, when considering OCC-specific survival rate, recurrence-free survival rate, and SPC-free survival rate, no specific trends were observed in the YNSF group (all *P* > .05, Fig. 3). We compared the OS rate according to detailed factors by selecting control groups based on determinants of the YNSF group, that is, smoking, sex, and age. Consequently, we analyzed the effect of smoking, sex, and age by comparing the survival rate of the YNSF group with those of YNSF (n = 11), young never smoker males (n = 14), and old never smoker females (n = 86), but there were no differences in any subgroup analyses (all *P* > .05, Fig. 4).

## 4 Discussion

The global incidence of OCC was 3.8% of all cancer cases in 2012 and is estimated to increase to 62% over the next two decades.^[23]^ OCC is very common in South–Central Asia, particular in India, and is thought to be associated with betel nut chewing and tobacco/alcohol use. Although the proportion of YNSF, an unusual group without a high risk of OCC, is also increasing worldwide, epidemiological studies and risk factor analyses of this group in Asia are very rare, despite this being the most significant endemic area of OCC. We, therefore, examined the frequency of presentation of YNSF and the characteristics of these patients during the last decade in a node hospital that treats most OCC patients in Far East Asia. In this study, the annual rate of increase in the number of all OCC patients was remarkable, although the proportion of YNSF and females was not relatively high. This might be because patients who visit a single institution do not represent a change in prevalence across the whole country, and there may be a selection bias due to the confined subjects who are also treated in the same hospital.

Our study did not reveal preoperative characteristics specific to YNSF and therefore did not find any significant etiologies. As mentioned in the Introduction, the etiological value of HPV in OCC remains arguable, perhaps because the exact distinction of primary origin between the palatine tonsil and the base of tongue is difficult to ascertain in case of advanced stage of cancer located on the lateral border of the tongue. Wu and colleagues reported that poor oral hygiene might be related to OCC development in young never smoker patients^[24]^; however, there was no significant association between dental status and OCC in YNSF in our study. In addition to the oral hygiene status, orthodontic appliances commonly used by young females are expected to affect OCC in YNSF, although this could not be confirmed. Furthermore, in line with increasing interest in the oral microbiome, there has been a recent report that periodontal microbiota may be related to OCC development in HPV-negative non-smokers.^[25]^ In the same vein, we anticipated that the use of drugs such as steroids or immunosuppressants, possibly affecting oral immunity against cancer, would be associated with OCC in YNSF; however, only eight (2.3%) patients overall were receiving such drugs, and no comparisons could thus be made.

In our study, we did not identify clinicopathological characteristics or survival trends specific to OCC in YNSF. In their study of OCC in non-smokers, Durr and colleagues reported that the patients showed a greater DOI and more advanced stage, resulting in lower OS rates.^[11]^ In contrast, recent reports, including a systematic review, demonstrated that the prognostic factors of young OCC patients were not different from those of old patients.^[6,14]^ Similarly, the results of genetic and molecular analyses confirmed that there is no difference in molecular expression and subsequent mechanism of carcinogenesis between the young and old patients.^[10]^ However, a recent study of comprehensive molecular profiling of 396 HNC patients who underwent radical surgery at a single institution reported that programmed death-ligand 1 (PD-L1) expression in tumor cells was high in young patients, and PD-L1 positivity and high tumor infiltrating CD163^+^ macrophage-to-CD8^+^ lymphocyte ratio may predict poor progression-free survival.^[26]^ Hence, it is necessary to analyze the association between antitumor immunity and biological characteristics of OCC in YNSF through comprehensive immunological profiling. Finally, we compared subgroups to examine the effects of smoking, sex, and age, which form the definition of YNSF; however, no significant differences in OS based on these characteristics were identified. A recent report in Australia demonstrated that old never smoker females with OCC showed lower survival rates than general OCC patients,^[27]^ which is contrary to the trend reported in the present study.

Our study included retrospective analysis of data from a single hospital and consequently cannot represent all the trends and characteristics of OCC patients in this country or region. In addition, because no histopathological analysis was conducted, this study is limited by the absence of marker analyses including molecular characteristics and mechanism of OCC development in YNSF. And there was no quantified objective measurement of oral health status such as oral hygiene index, consequently, the effect of oral hygiene on the occurrence of OCC in YNSF was not clearly identified in our study. However, this study is of value as the first attempt to investigate the epidemiological changes and clinicopathological characteristics of YNSF in Asia. To overcome the limitations of our study and to identify the etiologies and characteristics of OCC in YNSF, further investigations using large-scale cohort data to evaluate the prevalence of YNSF and molecular analyses focusing on the tumor immune microenvironment are warranted.

## 5 Conclusion

There has been no significant change in the annual incidence of YNSF among OCC patients followed at our institution over the last decade. In addition, no differences were found in either the clinicopathological characteristics or the survival trends of YNSF compared with those of other OCC patients.

## Author contributions

**Conceptualization:** Minsu Kwon, Yoon Se Lee.

**Data curation:** Minsu Kwon, Dong Kyu Lee.

**Formal analysis:** Minsu Kwon.

**Funding acquisition:** Yoon Se Lee.

**Investigation:** Minsu Kwon, Yoon Se Lee.

**Methodology:** Minsu Kwon, Dong Kyu Lee, Seung-Ho Choi, Soon Yuhl Nam, Sang Yoon Kim, Yoon Se Lee.

**Project administration:** Minsu Kwon.

**Resources:** Minsu Kwon.

**Software:** Minsu Kwon.

**Supervision:** Minsu Kwon, Yoon Se Lee.

**Validation:** Minsu Kwon, Yoon Se Lee.

**Visualization:** Minsu Kwon, Yoon Se Lee.

**Writing – original draft:** Minsu Kwon, Yoon Se Lee.

**Writing – review & editing:** Minsu Kwon, Dong Kyu Lee, Seung-Ho Choi, Soon Yuhl Nam, Sang Yoon Kim, Yoon Se Lee.
